# The Unfinished Business Scale for Families: A Measure for Evaluating the Unfinished Business of Bereaved Family Members of Terminally Ill Patients with Cancer in Japan

**DOI:** 10.1089/pmr.2024.0070

**Published:** 2025-05-13

**Authors:** Sakiko Matsuzaka, Mitsunori Miyashita, Kento Masukawa, Hiroyuki Otani, Tatsuya Morita, Masanori Mori

**Affiliations:** ^1^ Department of Palliative Nursing, Health Sciences, Tohoku University Graduate School of Medicine, Sendai, Japan.; ^2^ Palliative Care Team and Palliative and Supportive Care, National Hospital Organization Kyushu Cancer Center, Fukuoka, Japan.; ^3^ Department of Palliative and Supportive Care, Seirei Mikatahara General Hospital, Shizuoka, Japan.

**Keywords:** family, measurement tool, neoplasms, palliative care, unfinished business

## Abstract

**Objectives:**

Although unfinished business is associated with psychological states in bereaved families of patients with cancer, no evaluation tools have been developed for such families in Japan. This study aimed to develop and examine the validity and reliability of an evaluation tool for unfinished business among families of terminally ill patients with cancer in Japan.

**Methods:**

In August 2020, a cross-sectional online survey consisting of the Unfinished Business Scale for Families, Unfinished Business in Bereavement Scale (UBBS), Brief Grief Questionnaire (BGQ), and Patient Health Questionnaire-9 (PHQ-9) was conducted on bereaved families of patients with cancer, followed by a retest two weeks later.

**Results:**

Responses from 206 bereaved families were analyzed using factor analysis. Three subscales (10 items) were identified: Talk, Action, and Message. The Unfinished Business Scale for Families had an overall Cronbach’s α coefficient of 0.96, and the intraclass correlation coefficient in the test–retest examination was 0.74. The Unfinished Business Scale for Families was significantly moderately correlated with the UBBS (*r* = 0.46) and moderately correlated with the BGQ (*r* = 0.40) and PHQ-9 (*r* = 0.33). All *p*-values were <0.001.

**Conclusions:**

Our findings suggest that the Unfinished Business Scale for Families is effective for evaluating unfinished business among families of terminally ill patients with cancer. In the future, it will be necessary to conduct bereaved family surveys using this scale to identify unfinished business among family members. This could lead to health care providers providing more appropriate and adequate care to families with unfinished business.

## Introduction

Unfinished business, which is defined as “incomplete, unexpressed, or unresolved relationship issues with the deceased”^1,2^ or a “cognitive process that involves appraising one’s relationship with a deceased loved one as incomplete, unexpressed, or unresolved, lacking closure,^1^” can affect the psychological states of family members of terminally ill patients with cancer. Previous studies have reported that 26%–43% of such bereaved families might have unfinished business, and that about 70% have moderate or higher levels of distress related to this unfinished business.^2,3^ Other studies have found that distress related to unfinished business, such as depression and complicated grief (CG), is associated with psychological states in bereaved families of patients with cancer.^2–4^ Therefore, assessing unfinished business may help reduce levels of depression and CG in bereaved families.

To assess the degree of unfinished business in families of patients with cancer, a measurement tool known as the Unfinished Business in Bereavement Scale (UBBS) was previously developed in the United States.^5^ The UBBS examines unfinished business from two dimensions: Unfulfilled Wishes (feeling a sense of incompleteness with the deceased) and Unresolved Conflicts (unresolved issues between the family and the deceased).^5^ Using such a measurement tool to assess unfinished business from various dimensions could help clinicians understand what kinds of unfinished business families of patients with cancer possess and identify families at high risk for resultant depression and CG.

However, no tool has been developed to evaluate unfinished business in bereaved families of patients with cancer in Japan. In addition, no studies have examined how many families experience unfinished business using tools with assured reliability and validity. As the UBBS was developed in the United States, its contents are not suitable for Japanese people because of the numerous social and cultural differences between the two countries. Moreover, the UBBS does not include the contents related to unfinished business identified in previous studies in Japan, such as dialogue with a loved one.^3^ Japan is a high-context communication culture country, so Japanese people do not find direct communication necessary. Thus, the concept of unfinished business in Japan might differ from that in the United States and have a stronger impact. Given this background, the present study aimed to develop and examine the validity and reliability of a tool to evaluate unfinished business among bereaved families of patients with cancer in Japan.

## Methods

### Study design

This cross-sectional online survey was approved by the institutional review board of Seirei Mikatahara General Hospital (No. 20-23).

### Participants

Bereaved family members who met the following criterion were recruited from an Internet panel of a market research company (Macromill Inc., Tokyo, Japan): aged ≥18 years and had experienced the loss of a patient with cancer within the preceding five years.

### Procedures

In August 2020, we conducted two anonymous online surveys of bereaved family members of terminally ill patients with cancer in Japan. The first online survey was conducted to examine factor validity, internal consistency, concurrent validity, and discriminate validity. The follow-up online survey was conducted on the same participants two weeks after the first to examine test–retest reliability.

In the first survey, participants were recruited from an Internet panel of a market research company by sending an invitation to all registered monitors. The invited participants were screened by having them answer various questions, and those who met the eligibility criteria were included in the study. Bereaved families who consented to participate in the study then completed a questionnaire, after which, they were asked whether they could participate in the second survey.

In the second survey, the participants who responded to the first were invited in the same way as for the initial survey. The second survey was completed when the number of respondents reached the expected number.

### Measurements

#### Unfinished Business Scale for families

We conducted a literature review and discussed the contents of this scale with other researchers. Based on previous studies regarding unfinished business among bereaved families in Japan,^3,6,7^ we identified the following three subscales of unfinished business: Talk, Action, and Message. We also identified 16 potential unfinished business attributes. These attributes were graded on a seven-point Likert-type scale, from 1 = absolutely disagree to 7 = absolutely agree. Additionally, two simple items were included: one about whether the family had any unfinished business (again scored on a seven-point Likert-type scale, from 1 = absolutely disagree to 7 = absolutely agree), with higher scores indicating more unfinished business, and the other about the level of distress related to unfinished business over the preceding month (scored on a four-point Likert-type scale, from 1 = not at all distressed to 4 = extremely distressed), with higher scores indicating more distress. Face validity was evaluated by four people (one bereaved family member, one clinician, and two bereaved family members with professional medical licenses). This process ensured the content validity of our questionnaire.

#### Unfinished Business in Bereavement Scale (short version)

The short version of the UBBS was developed in the United States to assess unfinished business in families of patients with cancer.^5^ In the present study, we used a total of eight items, consisting of four items each from the two dimensions of Unfulfilled Wishes and Unresolved Conflicts. The Japanese version of the UBBS was created using the forward/backward translation method. The participants were asked to rate the level of distress associated with each unfinished business item on a five-point scale (from 1 = not at all distressed to 5 = extremely distressed), with higher scores indicating more unfinished business.

To examine concurrent validity, the following scales were used.

##### Brief Grief Questionnaire

We used the Brief Grief Questionnaire (BGQ) to assess CG.^8^ Both the reliability and validity of this scale have been confirmed in Japan.^9^ The BGQ is composed of five items, each rated on a scale from 0 to 2, with higher scores indicating a more severe grief reaction. The scores were then converted to a 0–10 point scale. A total score ≥8 indicated that the respondent was likely to develop CG.

##### Patient Health Questionnaire-9

The Patient Health Questionnaire-9 (PHQ-9) is composed of nine items used to assess the severity of depression.^10^ The reliability and validity of this scale have been confirmed in Japan.^11^ The scores were then converted to a 0–27 point scale. A total score ≥10 was considered a valid cutoff point for moderate to severe depression.

#### Participant characteristics

We asked about the patient’s disease, the bereaved family member’s age, sex, relationship to the deceased, duration of bereavement, and frequency of caring for the patients.

### Statistical analyses

All statistical analyses were performed using SAS ver. 9.4, Japanese version (Cary, NC; BMDP, Los Angeles, CA). For item reduction, we first deleted attributes with 20% or more of the data missing or a highly skewed distribution of ratings, which was defined as “absolutely disagree” or “absolutely agree” for 80% of the responses. We then performed explanatory factor analysis using principal component analysis with promax rotation. Based on the results of the factor analysis, items with a factor loading <0.4 were deleted. In addition, we discussed the final adoption of items with regard to exhaustibility and clinical viewpoints. We also examined whether each item’s response distribution was strongly skewed (e.g., a large ceiling or floor effect).

To examine the validity of the Unfinished Business Scale for Families, we first computed correlations between each subscale to assess construct validity. Second, we calculated Pearson’s correlation coefficients between each subscale and the UBBS, BGQ, and PHQ-9 to assess concurrent validity. Because both the Unfinished Business Scale for Families and UBBS are measures of family members’ unfinished business, we hypothesized the presence of a moderate to high correlation between them. We also hypothesized the presence of a low to moderate correlation between the Unfinished Business Scale for Families and both the PHQ and BGQ, because of the associations between having unfinished business with family members and bereaved outcomes such as depression and CG. The correlation coefficients were defined as 0–0.3 for a small correlation, 0.3–0.5 for a moderate correlation, and 0.5–1.0 for a strong correlation.

Cronbach’s α coefficient was used to examine internal consistency for all attributes and subscales, and the intraclass correlation coefficient (ICC) to assess test–retest reliability.

## Results

The study participants were 206 bereaved family members of a patient who had died from cancer. Among these participants, 106 participated in the second survey to assess test–retest reliability.

### Participant characteristics

All responses from the 206 bereaved family members of terminally ill patients with cancer were subjected to analysis. Table 1 summarizes the backgrounds of the patients and their family members.

**Table 1.** Backgrounds of the Patients and Bereaved Families

Patients		
Disease, *n* (%)		
Lung	43	(20.9)
Stomach, esophagus, colon, rectum	58	(28.1)
Liver, bile duct, pancreas	41	(19.9)
Breast	13	(6.3)
Prostate, kidney, bladder	10	(4.9)
Ovary, uterus	8	(3.9)
Blood	17	(8.3)
Other	16	(7.8)
Bereaved families		
Age, average (SD)	48.8	(13.2)
Sex, *n* (%)		
Male	98	(47.6)
Female	108	(52.4)
Relationship to the deceased, *n* (%)		
Spouse	6	(2.9)
Child	72	(35.1)
Son/daughter-in-law	10	(4.9)
Parent	14	(6.8)
Brother/sister	28	(13.7)
Other	75	(36.6)
Duration of bereavement, *n* (%)		
<1 year	41	(19.9)
1–3 years	92	(44.7)
3–5 years	72	(34.3)
>5 years	1	(0.5)
Frequency of caring for patients, *n* (%)		
Every day	41	(19.9)
4–6 days/week	21	(10.2)
3 days/week or less	50	(24.3)
Not at all	86	(41.8)

SD, standard deviation.

### Frequency distribution of responses to the Unfinished Business Scale for Families

Table 2 shows the frequency distribution of responses to the Unfinished Business Scale for Families. In total, 44.8% (*n* = 92) of the bereaved families responded that they had unfinished business regarding the last weeks with their patient and 64.6% that they had distress related to unfinished business within the past month.

**Table 2.** Unfinished Business Scale for Families (Frequency Distribution)

		*N* (%)						
	Mean (SD)	Absolutely disagree	Disagree	Somewhat disagree	Unsure	Somewhat agree	Agree	Absolutely agree
1. I wish I had talked about more things with the patient.	5.0 (0.4)	3 (1.5)	17 (8.3)	11 (5.3)	41 (20.0)	59 (28.6)	40 (19.4)	35 (17.0)
2. I wish I had talked about more things with the assumption that he/she will die.	4.8 (1.6)	4 (1.9)	22 (10.7)	15 (7.3)	62 (30.1)	47 (22.8)	33 (16.0)	23 (11.2)
3. I wish I had listened more about the thoughts and actual feelings of the patient.	4.8 (1.4)	6 (2.9)	17 (8.3)	17 (8.3)	58 (28.2)	54 (26.2)	29 (14.1)	25 (12.1)
4. I wish I knew what the patient thought of me.	3.9 (1.6)	17 (8.3)	38 (18.5)	19 (9.2)	69 (33.5)	26 (12.6)	24 (11.7)	13 (6.3)
5. I wish the patient had left his/her thoughts, messages, and important things for me.	3.8 (1.4)	13 (6.3)	31 (15.1)	35 (17.0)	69 (33.5)	29 (14.1)	21 (10.2)	8 (3.9)
6. I wish I could have heard the gratitude and words of farewell from the patient.	3.6 (1.6)	18 (8.7)	42 (20.4)	37 (18.0)	59 (28.6)	30 (14.6)	8 (3.9)	12 (5.8)
7. I wish I had said more about what I wanted to say.	4.6 (1.4)	8 (3.9)	19 (9.2)	15 (7.3)	68 (33.0)	43 (20.9)	34 (16.5)	19 (9.2)
8. I wish I had expressed the gratitude I had for the patient.	4.8 (1.5)	6 (2.9)	16 (7.8)	11 (5.3)	55 (26.7)	56 (27.2)	34 (16.5)	28 (13.6)
9. I wish I had done more things with the patient.	5.1 (1.4)	5 (2.4)	14 (6.8)	16 (7.8)	56 (27.2)	51 (24.8)	29 (14.1)	35 (17.0)
10. I wish I had done more things on the assumption that he/she will die.	4.7 (1.5)	5 (2.4)	17 (8.3)	20 (9.7)	63 (30.6)	48 (23.3)	25 (12.1)	28 (13.6)
11. I wish we had spent more time together.	4.8 (1.5)	3 (1.5)	16 (7.8)	14 (6.8)	55 (26.7)	53 (25.7)	33 (16.0)	32 (15.5)
12. I wish I had known more about the patient’s wishes after death (e.g., funeral or putting his/her affairs in order).	4.2 (1.6)	10 (4.9)	25 (12.1)	21 (10.2)	88 (42.7)	36 (17.5)	17 (8.3)	9 (4.4)
13. I wish I could have helped the patient fulfill what he/she wanted to do.	4.6 (1.4)	5 (2.4)	16 (7.8)	13 (6.3)	62 (30.1)	54 (26.2)	30 (14.6)	26 (12.6)
14. I wish I could have helped the patient meet the person he/she wanted to see.	4.5 (1.4)	6 (2.9)	15 (7.3)	14 (6.8)	83 (40.3)	42 (20.4)	29 (14.1)	17 (8.3)
15. I wish I could have done something special for the patient.	4.5 (1.5)	8 (3.9)	18 (8.7)	13 (6.3)	77 (37.4)	45 (21.8)	25 (12.1)	20 (9.7)
16. I wish I could have been with the patient at the moment of death.	4.8 (1.6)	9 (4.4)	17 (8.3)	11 (5.3)	64 (31.1)	40 (19.4)	27 (13.1)	38 (18.5)
Overall, do you have any “unfinished business” concerning the final few weeks you spent with the patient?	4.5 (1.6)	21 (10.2)	27 (13.1)	22 (10.7)	44 (21.4)	58 (28.2)	17 (8.3)	17 (8.3)

	Mean (SD)	*N*			
		Not at all distressed	Somewhat distressed	Distressed	Extremely distressed
How distressed you have been about this issue in the past month?	2.4 (1.0)	73 (35.4)	79 (38.4)	39 (18.9)	15 (7.3)

SD, standard deviation.

### Factor analysis

Item analysis revealed no large ceiling or floor effects. In accordance with the above-mentioned item-reduction procedure, three subscales and 10 items were selected. The final results of the factor analysis for all subscales are shown in Table 3.

**Table 3.** Factor Validity of the Unfinished Business Scale for Families

	Standardized regression coefficients			
	Factor 1	Factor 2	Factor 3	Communality
Talk subscale (ICC = 0.69, α = 0.91)				
I wish I had talked about more things with the patient.	**0.70**	0.30	–0.01	0.74
I wish I had done more things on the assumption that he/she will die.	**0.86**	–0.10	0.09	0.71
I wish I had expressed the gratitude I had for the patient.	**0.50**	0.45	–0.01	0.74
I wish I had listened more about the thoughts and actual feelings of the patient.	**0.73**	0.14	0.12	0.81
Action subscale (ICC = 0.7, α = 0.98)				
I wish I could have helped the patient fulfill what he/she wanted to do.	0.13	**0.78**	–0.03	0.72
I wish I could have helped the patient meet the person he/she wanted to see.	–0.05	**0.86**	0.10	0.79
I wish I could have done something special for the patient.	0.12	**0.64**	0.18	0.71
Message subscale (ICC = 0.63, α = 0.87)				
I wish I could have heard the gratitude and words of farewell from the patient.	–0.13	0.12	**0.80**	0.65
I wish the patient had left his/her thoughts, messages, and important things for me.	0.11	0.08	**0.79**	0.82
I wish I knew what the patient thought of me.	0.19	–0.07	**0.74**	0.67
Total score of the Unfinished Business Scale for Families (ICC = 0.71, α = 0.96)				

ICC, intraclass correlation coefficient.

Boldface indicates factor loadings ≥ 0.40 on the designated primary factor of each subscale.

### Internal consistency and reliability

Table 3 also shows the results regarding the internal consistency of the Unfinished Business Scale for Families. Cronbach’s α ranged from 0.87 to 0.91 for all subscales and the ICCs from 0.63 to 0.74.

### Construct validity

Table 4 shows the correlations between the subscales of the Unfinished Business Scale for Families. Strong correlations were found between the total scale score and that of each subscale, and moderate to strong correlations were found between the subscales.

**Table 4.** Spearman’s Correlation Coefficients

	Talk subscale	Action subscale	Message subscale
Subscale			
Talk	1.00	0.75*	0.61*
Action	0.75*	1.00	0.61*
Message	0.61*	0.61*	1.00
Unfinished business in the last few weeks	0.58*	0.51*	0.43*
Distress related to unfinished business	0.32*	0.31*	0.31*
Total score of the Unfinished Business Scale for Families	0.92*	0.88*	0.78*

*p < 0.001.

### Concurrent validity

Table 5 shows the results regarding concurrent validity. We calculated Pearson’s correlation coefficients between the Unfinished Business Scale for Families and the UBBS, BGQ, and PHQ-9. The Unfinished Business Scale for Families was moderately correlated with the UBBS (*r* = 0.46, *p* < 0.001) and moderately correlated with the BGQ (*r* = 0.40, *p* < 0.001) and PHQ-9 (*r* = .33, *p* < 0.001). The correlations between the UBBS and BGQ and the PHQ-9 were *r* = 0.59 (*p* < 0.001) and *r* = 0.44 (*p* < 0.001), respectively.

**Table 5.** Concurrent Validity, Spearman’s Correlation Coefficient

	Total score of UBBS	Unfulfilled wishes	Unresolved conflict	BGQ	PHQ-9
Subscale					
Talk	0.35*	0.46*	0.19*	0.28*	0.23**
Action	0.42*	0.50*	0.29*	0.38*	0.32*
Message	0.49*	0.50*	0.40*	0.38*	0.37*
Total score of the Unfinished Business Scale for Families	0.46*	0.55*	0.31*	0.40*	0.33*

*p < 0.001; **p < 0.05.

BGQ, Brief Grief Questionnaire; PHQ-9, Patient Health Questionnaire-9; UBBS, Unfinished Business in Bereavement Scale.

## Discussion

In the present study, we developed an instrument to assess unfinished business among bereaved family members of terminally ill patients with cancer. Construct validity was confirmed by explanatory factor analysis. The Unfinished Business Scale for Families has three subscales: Talk, Action, and Message. The Talk and Action subscales express regret about the talking and action the bereaved family member felt that they should have done with the dying patient. The Message subscale expresses regret about messages the bereaved family member had wanted to exchange with the dying patient. This subscale included not only items related to messages from the patient to the family but also an item regarding the patients’ thoughts of their bereaved families: “I wish I knew what the patient thought of me.” These subscales are not consistent with the UBBS developed in the United States. The UBBS consists of two subscales: Unfulfilled Wishes and Unresolved Conflicts.^5^ The subscales of the Unfinished Business Scale for Families are similar to the Unfilled Wishes subscale of the UBBS. This indicates that this concept is similar between the United States and Japan. However, the themes of the UBBS were created from qualitative studies of unfinished business in the United States. On the contrary, the Unfinished Business Scale for Families used items on unfinished business developed from previous studies on the achievement of a good death in Japan and the family’s end-of-life experience.^12–14^ The UBBS and Unfinished Business Scale for Families have different structures because of the different references used for their development. Therefore, the Unfinished Business Scale for Families is considered to be appropriate for Japanese culture. However, it should be noted that the item “I wish I had expressed the gratitude I had for the patient” of the Talk subscale may belong to both the Talk and Action subscales. Future studies should evaluate the construct validity of the scale in regard to this point.

The concurrent and discriminant validity of the Unfinished Business Scale for Families was reasonable. The scale was moderately correlated with the UBBS, which evaluates the same concept of unfinished business. We hypothesized that the Unfinished Business Scale for Families would be more strongly associated with the UBBS because they both evaluate the unfinished business of families. This result might have been caused by the difficulties sometimes encountered by the Japanese in answering items on the Japanese version of the UBBS because it consists of questions specific to the United States. As mentioned above, the Unfinished Business Scale for Families and UBBS have different compositions; therefore, it is significant that we have created a scale that can accurately assess unfinished business in Japan.

Additionally, the Unfinished Business Scale for Families was weakly correlated with both the BGQ and PHQ-9. Previous studies have also reported that unfinished business is associated with depression and grief among bereaved families.^3,15^ This finding suggests that reducing unfinished business among family members might help prevent depression and CG among bereaved families. Therefore, the ability to assess unfinished business using a reliable and validated scale is important for reducing depression and grief among bereaved families.

The reliability of the Unfinished Business Scale for Families was confirmed based on excellent internal consistency (Cronbach’s α coefficient = 0.87–0.98) and fair test–retest reliability (ICC = 0.63–0.7). The reliability of the total score was high, but the reliability of each subscale was moderate. This result was considered to have been caused by recall bias. As the present survey was conducted on families after bereavement, the possibility of recall bias cannot be ignored. Therefore, the ICCs of the Unfinished Business Scale for Families were considered acceptable.

This study had several limitations. First, the sample size was small, and the average age of the sample was younger than the general population of bereaved family members of terminally ill patients with cancer because the participants in the present study were all bereaved family members who had been registered with a market research company. Therefore, our study sample may not be representative of the general population in Japan. Second, recall bias may have affected the results because of the duration between the death of the patient and participation in the survey. Third, we did not collect information on the educational attainment or income level of bereaved family members, both of which might impact grief. Fourth, as this study was conducted in Japan, the results may not be applicable to other countries. Larger studies are needed to explore the frequency and associated factors of unfinished business by utilizing the Unfinished Business Scale for Families in various clinical settings (e.g., palliative care units, palliative care teams) in Japan and other countries. Moreover, future efforts should be made to develop care strategies for terminally ill patients and their families to reduce unfinished business and related distress. The present scale could serve as an important outcome measure in future prospective studies evaluating the effectiveness of such care strategies.

In conclusion, the Unfinished Business Scale for Families appears to be a useful tool with sufficient validity and reasonable reliability for measuring unfinished business among bereaved families in Japan. The results of this study could be expected to improve and provide a better understanding of the current status of bereaved family members of terminally ill patients with cancer. In the future, it will be necessary to conduct bereaved family surveys using the present scale to identify unfinished business among family members. This could lead to health care providers providing more appropriate and adequate care to families with unfinished business.

## Authors' Contributions

S.M.: Conceptualization and study design, data analysis, interpretation ofresults, article writing with draft preparation, and revision. K.M.: Conceptualization and study design, data analysis, interpretation ofresults, article writing, and revision. M. Miyashita., H.O., T.M., and M. Mori.: Conceptualization and study design, data collection, acquisition, interpretation of results, article writing, and revision.

## Abbreviations Used

BGQ: Brief Grief Questionnaire
CG: complicated grief
PHQ-9: Patient Health Questionnaire-9
SD: standard deviation
UBBS: Unfinished Business in Bereavement Scale
